# Performance analysis of novel methods for detecting epistasis

**DOI:** 10.1186/1471-2105-12-475

**Published:** 2011-12-15

**Authors:** Junliang Shang, Junying Zhang, Yan Sun, Dan Liu, Daojun Ye, Yaling Yin

**Affiliations:** 1 School of Computer Science & Technology, Xidian University, Xi'an 710071, China; 2 Shannxi people's fine arts publishing house, Xi'an 710003, China; 3 Information School, Xi'an Economical and Financial University, Xi'an 710100, China

## Abstract

**Background:**

Epistasis is recognized fundamentally important for understanding the mechanism of disease-causing genetic variation. Though many novel methods for detecting epistasis have been proposed, few studies focus on their comparison. Undertaking a comprehensive comparison study is an urgent task and a pathway of the methods to real applications.

**Results:**

This paper aims at a comparison study of epistasis detection methods through applying related software packages on datasets. For this purpose, we categorize methods according to their search strategies, and select five representative methods (TEAM, BOOST, SNPRuler, AntEpiSeeker and epiMODE) originating from different underlying techniques for comparison. The methods are tested on simulated datasets with different size, various epistasis models, and with/without noise. The types of noise include missing data, genotyping error and phenocopy. Performance is evaluated by detection power (three forms are introduced), robustness, sensitivity and computational complexity.

**Conclusions:**

None of selected methods is perfect in all scenarios and each has its own merits and limitations. In terms of detection power, AntEpiSeeker performs best on detecting epistasis displaying marginal effects (eME) and BOOST performs best on identifying epistasis displaying no marginal effects (eNME). In terms of robustness, AntEpiSeeker is robust to all types of noise on eME models, BOOST is robust to genotyping error and phenocopy on eNME models, and SNPRuler is robust to phenocopy on eME models and missing data on eNME models. In terms of sensitivity, AntEpiSeeker is the winner on eME models and both SNPRuler and BOOST perform well on eNME models. In terms of computational complexity, BOOST is the fastest among the methods. In terms of overall performance, AntEpiSeeker and BOOST are recommended as the efficient and effective methods. This comparison study may provide guidelines for applying the methods and further clues for epistasis detection.

## Background

Compared with Mendelian diseases, complex diseases, i.e., non-Mendelian diseases, represent the major part of diseases in human and other model organisms [1], such as Alzheimer's disease, cancer, heart disease, type 2 diabetes and many others. They are supposed to be caused by multiple single nucleotide polymorphisms (SNPs), their interactive effects, and/or their interactions with environmental factors [2-4]. The interactive effects of multiple SNPs underlying complex diseases are often referred to as epistasis or epistatic interactions [5,6]. It is now believed to be one of the causative patterns of complex diseases [7]. There is a wide spectrum of epistasis. Some show both marginal effects and interactive effects and others show no marginal effects but interactive effects [8-10]. We refer to the former as epistasis displaying marginal effects (eME) and the latter as epistasis displaying no marginal effects (eNME). Epistasis detection is to explore all the epistasis including both eME and eNME from a dataset for genome-wide association studies (GWAS). In fact, detection of epistasis and characterization of the effects of those epistatic interactions are both a goal and a challenge [11].

For identifying epistasis in biological datasets, some pioneering work has been reported. For small scale datasets, exhaustive methods, including combinatorial partitioning method (CPM) [12], multifactor dimensionality reduction (MDR) [13], restricted partitioning method (RPM) [14], information gain (IG) [15] and backward genotype-trait association (BGTA) [16], appear promising, though most of them have not been validated yet in their effectiveness for large scale datasets. Recently many stochastic and heuristic methods have been developed [7,8,17-26] in GWAS, which may retain as many informative SNPs as possible while largely reducing computational complexity [27]. For example, Tang *et al *proposed epistatic module detection (epiMODE) [7], which is a generalized method of Bayesian epistasis association mapping (BEAM) [8]. Wang *et al *used AntEpiSeeker [17] to identify epistasis, which is a two-stage ant colony optimization algorithm (ACO). Wan *et al *proposed SNPRuler [18] based on both predictive rule inference and two-stage design. They also proposed another method, Boolean operation-based screening and testing (BOOST) [19], which involves only Boolean values and allows the use of fast logic operations to obtain contingency tables. Zhang *et al *proposed a series of methods [20-23], which exploit some properties of test statistic to mitigate multiple testing problems. Among them, Tree-based epistasis association mapping (TEAM) [23] updates contingency tables of two-locus tests by utilizing a minimum spanning tree.

Although almost all methods in their respective articles are demonstrated as computationally and statistically useful tools in the coming era of large scale interaction mapping and several review articles [11,28-30] and web pages [31,32] appear, unfortunately, their performance in common datasets remains largely unclear. Till now, there have been few studies focused on in-depth independent comparison of the methods [27,33-40]. Ritchie *et al *[33] examined the detection power of MDR in the presence of noise due to genotyping error, missing data, phenocopy and genetic heterogeneity. Motsinger-Reif *et al *[34] used the same criteria to compare the performance of grammatical evolution neural network and MDR. Both studies did not consider sensitivity and computational complexity, which are critical to large scale datasets. Chen *et al *[27,35] executed comparative studies based on ground-truth SNPs and designed a series of evaluation criteria. Fritsch *et al *[36] compared the performance of four regression based methods in both simulation and real datasets. He *et al *[37] assessed the performance of MDR and penalized logistic regression method on models with different magnitudes of interactive effects under the criteria of log-odds, prediction error and detection power. More recently, Wang *et al *[38] evaluated five novel methods in terms of detection power, type-1 error rate, scalability and completeness. In these articles, noise was not considered, which is often presented in biological datasets and may affect results of methods severely.

Lack of benchmark simulation datasets, limited epistasis models, evaluation criteria and computational complexity are main difficulties for comparison study. Currently, different methods are evaluated in different datasets simulated by diverse tools; most of epistasis models are based on weak theories of biological systems (e.g., a complex disease may not be caused by only a simple mathematical model, such as XOR [10], ZZ [41], dominant, additive and recessive models [42]); existing evaluation criteria may not be sufficiently objective; computational burden imposed by enormous search space is intensive. All the above are the great challenges in association studies, especially in GWAS.

The goal of this study is to reveal performance of selected methods and provide guidelines for applying them. By reviewing the literature, 36 methods in use are identified. We then classify these methods into three categories according to their search strategies and select five representative methods for comparison. They are TEAM, BOOST, SNPRuler, AntEpiSeeker and epiMODE. Diverse performance evaluation criteria are provided, including detection power (three forms are introduced), robustness, sensitivity and computational complexity. Experiments are performed on simulation datasets, which are with different size, various epistasis models, and with/without noise. Three types of noise, i.e., missing data, genotyping error and phenocopy, are considered in the experiments.

## Methods

**Figure 1:**
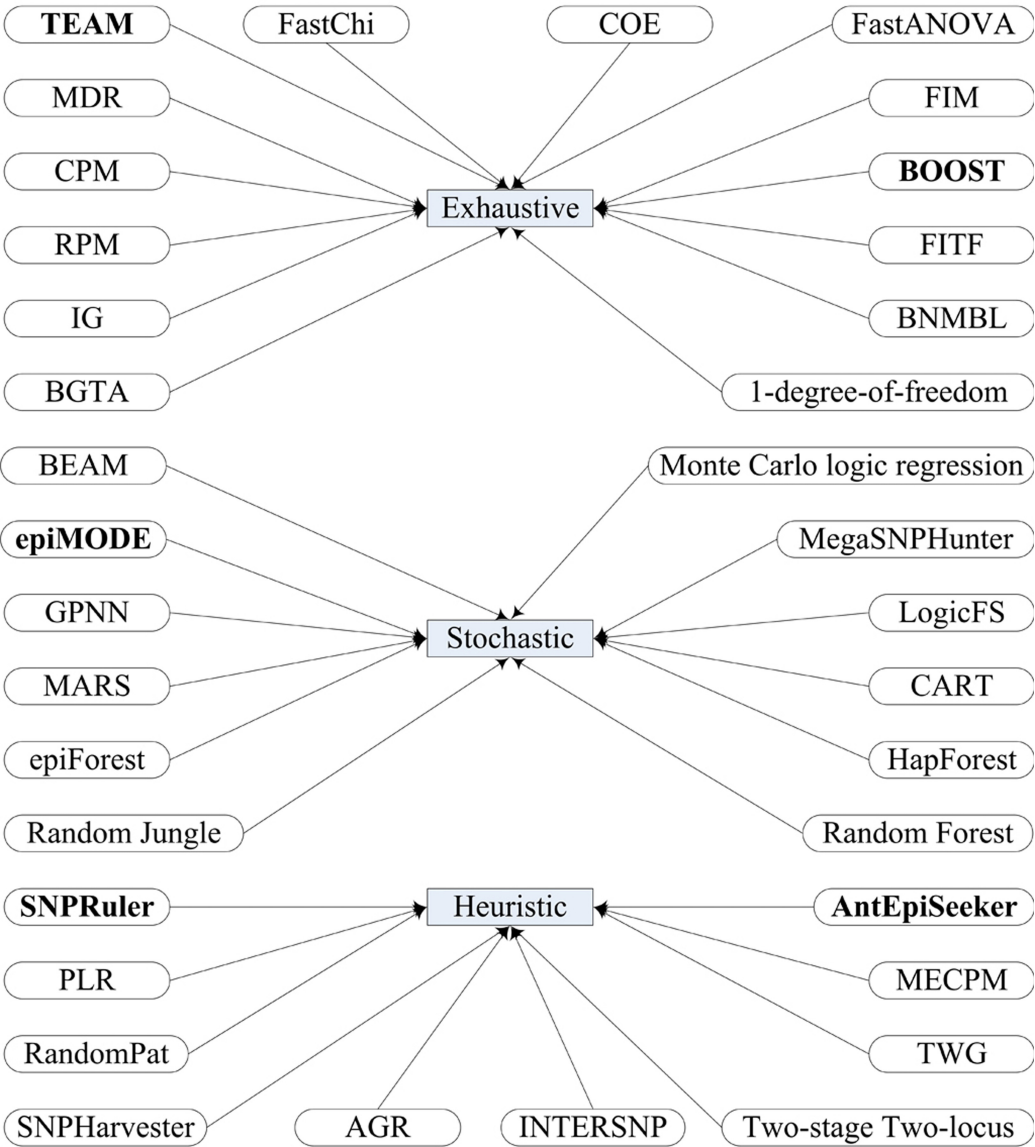
**Classification of the methods that detect epistasis**. All methods can be classified into three categories according to their search strategies, i.e., exhaustive search, stochastic search, and heuristic search. Methods with bold names are described and evaluated in detail. Detailed information of these methods is provided in supplementary table S1 in additional file 1.

In a review of the literature, we identify 36 methods in use for detecting epistasis, excluding specializations and tweaks [43]. An overview of these methods is depicted in Figure 1, with details provided in supplementary table S1 in additional file 1. From Figure 1, one can see that the methods can be classified into three categories according to their search strategies, i.e., exhaustive search, stochastic search and heuristic search. Exhaustive search enumerates all *K*-locus interactions among SNPs to identify the effect or effects that best predict the phenotype. It prohibits application to GWAS on identifying high-order interactions since its combinatorial explosion of running time with respect to the interaction order of SNPs. Stochastic search performs a random investigation of search space and its performance relies on random chance to select phenotype-associated SNPs. With the number of SNPs growing, it is believed that the chances of correct guess dramatically drop. Heuristic search guarantees to obtain locally optimal solutions at the given conditions based on available information. It is likely to miss globally optimal solution, especially when it is an eNME.

### Methods being compared

It is unrealistic to comprehensively compare all 36 methods at affordable time cost. For this reason, we select five representative methods for our comparison study. The methods are recently proposed and claimed to facilitate large scale datasets and their packages are available online. They are TEAM, BOOST, SNPRuler, AntEpiSeeker and epiMODE (see original references [7,17-19,23] for their details). Their main similarities/differences are provided in supplementary table S2 in additional file 1. Below we introduce their respective principles briefly.

#### 1. TEAM

TEAM (Tree-based Epistasis Association Mapping) [23] exhaustively computes all two-locus interactions using permutation test. Permutation test is generally more accurate than direct-adjustment methods (e.g., Bonferroni correction) in identifying significant epistatic interactions, but at a higher computational cost. Notice that if two SNPs have the same genotypes on most samples, the computation of their contingency tables can be shared by considering only those samples with different genotypes [38], TEAM utilizes a minimum spanning tree to maximize the computation sharing of contingency tables for reducing the computational cost, where a node represents a SNP and an edge weight denotes the number of samples with different genotypes between connected SNPs. Such a tree makes it faster than brute-force methods by an order of magnitude. Since permutation test is unable to differentiate eNME from eME [44], TEAM focuses on identifying epistasis including both eNME and eME.

#### 2. BOOST

BOOST (BOolean Operation-based Screening and Testing) [19] is a two-stage method. It examines all two-locus interactions in screening stage and the ones which pass a user-specified threshold are then tested in testing stage. In screening stage, interactive effect of a SNP pair is represented by Kullback-Leibler divergence $D=N\cdot D_{KL}\left(\hat{\pi }||\hat{p}\right)$ where $\hat{\pi }$ is the joint distribution estimated under the full logistic regression model $M_{S}=\beta_{0}+\beta_{i}^{x_{1}}+\beta_{j}^{x_{2}}+\beta_{ij}^{x_{1}x_{2}}$, and $\hat{p}$ is the approximate joint distribution estimated under the main logistic regression model $M_{H}=\beta_{0}+\beta_{i}^{x_{1}}+\beta_{j}^{x_{2}}$ using a method known as "Kirkwood superposition approximation". In testing stage, two statistic tests, i.e., likelihood ratio test and *chi*-squared test, are conducted to determine whether the interactive effect of a SNP pair is significant. BOOST is a model-based method that only focuses on identifying eNME. Its contributions to epistasis detection domain are the introduction of Boolean values to represent data and an upper bound of likelihood ratio test to prune insignificant epistatic interactions.

#### 3. SNPRuler

SNPRuler is a learning method based on predictive rule inference [18]. A predictive rule describing relationship between SNPs and the phenotype is applied to infer epistasis. Rule learning is carried out through a branch and bound search algorithm. In branch stage, SNPRuler builds a tree with a node representing a SNP and a path indicating a possible rule. Since exhaustive tree traversal is practically impossible due to the explosive number of combinations as the tree grows, relevance measure, i.e., an upper bound of *chi*-squared test, is introduced to quantify the importance of a path. Only the path with relevance measure higher than a user-specified threshold is retained. In bound stage, a post-procedure is used to order the retained paths by their relevance measures. These paths, i.e., the rules, are the final epistatic interactions. SNPRuler only tests eNME since it prunes eME in the branch stage [38].

#### 4. AntEpiSeeker

AntEpiSeeker [17] is a modified algorithm derived from the generic ACO [45]. It is also a two-stage method. In the first stage, *Chi*-squared test is used as a score function to measure the association between a *K*-locus interaction and the phenotype, and thus no assumption about the interaction is made in AntEpiSeeker. The probability of an ant adding SNP *k *into its path (i.e., a *K*-locus interaction) at iteration *i *is defined as $p_{k}\left(i\right)=\tau_{k}\left(i\right)/\overset{N}{\underset{j=1}{\sum }}\tau_{j}\left(i\right)$, where *τ_k_*(*i*) is the pheromone. The pheromone is updated according to $\tau_{k}\left(i+1\right)=\left(1-\rho \right)\cdot \tau_{k}\left(i\right)+0.1\cdot \overset{J}{\underset{j=1}{\sum }}\chi_{k}^{j}\left(i\right)$, where *ρ *is the evaporation coefficient, *J *is the number of *K*-locus interactions containing SNP *k *at iteration *i*, $\chi_{k}^{j}\left(i\right)$ is the *chi*-squared value of interaction *j*. In the second stage, AntEpiSeeker conducts an exhaustive search of interactions within the highly suspected SNP sets, and within the reduced set of SNPs with top ranking pheromone levels.

#### 5. EpiMODE

EpiMODE (epistatic MOdule DEtection) [7] is a generalized method of BEAM [8]. It introduces a notion of epistatic modules to describe interactive effects of multiple SNPs. An epistatic module is the smallest genetic unit that independently influences the phenotype. On the basis of the notion, finding SNPs having epistasis is equivalent to assigning SNPs to epistatic modules. The assignment is done by first calculating probability of observed data given a certain SNP partition pattern using a Bayesian model and then obtaining the posterior probability of a SNP belonging to each epistatic module. Gibbs sampling strategy with a reversible jump Markov chain Monte Carlo procedure is employed for the posterior probability. Finally, epiMODE resorts to hypothesis testing to screen out significant epistatic modules. Just like TEAM and AntEpiSeeker, epiMODE is also a method that focuses on both eME and eNME detection.

### Evaluation criteria

In our study, four criteria are used to evaluate the performance of a method.

Detection power is one of the generally used performance evaluation criteria in epistasis detection domain. Various forms of detection power have been proposed [7,8,18,24] depending on what is desired to measure. In this paper, three types of detection power with constraints ranging from conservative to modest are defined.

Before giving definitions of detection power, several terms and notations are introduced. A dataset refers to a collection of SNP data as well as the phenotype. The collection of SNP data is denoted as a matrix, in which a row represents genotypes of a sample and a column represents a SNP. The ground-truth SNPs, which are only applied to simulation datasets [27,35], refer to the causative SNPs that truly associated with the phenotype, i.e., the SNPs in models added into simulation datasets.

Since a complex disease may be caused by multiple epistatic interactions, each of which consists of one or more SNPs, it is necessary to simulate multiple epistasis models in a dataset. Suppose we generate *D *datasets with the same parameter settings for detection power calculation. For dataset *i*, let *S_i _*denote the number of independent epistasis models (i.e., no SNPs are involved in more than one model) and *k_ij _*be the number of SNPs involved in model *j*. Hence, the number of ground-truth SNPs in dataset *i *is $M_{i}=\overset{S_{i}}{\underset{j=1}{\sum }}k_{ij}$. In our experiments, a method returns a rank of SNPs implying their descending importance to the phenotype. We use the top *L_i _*SNPs and the *M_i _*ground-truth SNPs to define detection power.

*power 1 *is defined as the proportion of datasets in which all ground-truth SNPs are identified with no false positives. It is written as *power *$1=\frac{1}{D}\overset{D}{\underset{i=1}{\sum }}x_{i}$, where *x_i _*∈ {0,1} is the detection tag, i.e., if the detection set constituted by the top *L_i _*(*L_i _*= *M_i_*) SNPs includes all ground-truth SNPs in dataset *i*, *x_i _*= 1; otherwise, *x_i _*= 0.

*power 2 *is defined as an average proportion of true positives in the top *L_i _*(*L_i _*= *M_i_*) SNPs. It is written as *power *$2=\frac{1}{D}\overset{D}{\underset{i=1}{\sum }}\frac{y_{i}}{M_{i}}$, where *y_i _*is the number of ground-truth SNPs in the top *L_i _*(*L_i _*= *M_i_*) SNPs identified in dataset *i*.

*power 3 *is defined as the ratio of the number of ground-truth SNPs appearing in the top *L_i _*SNPs to *M*, and can be written as *power *$3=\frac{1}{D}\overset{D}{\underset{i=1}{\sum }}\frac{z_{i}}{M_{i}}$, where *z_i _*is the number of ground-truth SNPs in the top *L_i _*( *L_i _*>*M_i_*) SNPs detected in dataset *i*. In our experiment, *L_i _*is set to 4.

Robustness of a method is also measured. Though empirical and theoretical studies suggest that the methods have good performance on detection power, it is for non-noise datasets. The robustness of methods to noise remains unclear. For this study, we introduce "degree of robustness" (DOR) to quantify the robustness of a method on noise datasets. It is defined as a normalized relative decrease of detection power from non-noise datasets to noise datasets under unit degree of noise added into non-noise datasets in generating noise datasets. By setting $v=\left(\frac{P_{p}-P_{I}}{P_{p}+\varepsilon }\right)/I$, we define $T=\frac{2}{\sqrt{2\pi }}\int_{v}^{+\infty }e^{\frac{t^{2}}{2}}dt$ as the DOR of a method, where *P_p _*and *P_I _*are the detection power on non-noise datasets and noise datasets respectively, and *I *is the degree of noise added into non-noise datasets. In the definition, *ε *is introduced to avoid the denominator becoming zero. It is clear that the smaller *v *or equivalently larger *T *indicates stronger robustness.

Having been widely applied in references [27,35,46-48], receiver operating characteristic (ROC) curve is a graphical plot of the sensitivity versus false positive rate (FPR), showing how many ground-truth SNPs are detected for a given FPR. Since the number of SNPs meaningful to the phenotype is smaller compared to that of phenotype-unassociated SNPs, we measure sensitivity of a method at 0.01 FPR and show the left-side ROC curve as an intuitive evaluation [27].

Computational complexity is also considered. We measure running time in the same computational environment to assess realistic applicability of a method.

### Simulation Tool

We provide a tool, epiSIM, to simulate epistatic interactions in datasets for case-control association studies. EpiSIM offers several single-locus and epistasis models associated with the phenotype. It allows users to set parameters freely, including sample size, number of SNPs, variation range of minor allele frequencies (MAFs) in random SNPs, model types, linkage disequilibrium, penetrance functions, indexes of ground-truth SNPs, and so on, some of which jointly determine the strength of association between SNPs and the phenotype.

## Results and Discussion

### Detection Power Analysis

Though epistasis models have been widely discussed [49,50] and can be simulated by epiSIM, it is unrealistic to evaluate a method on all epistasis models with all possible parameter settings. In our experiments, we exemplify 9 commonly used two-locus epistasis models including three eME models and six eNME models for study. The first three epistasis models [7,8] are eME models. Their penetrance functions are shown in supplementary table S3 in additional file 1 and can be determined given population prevalence, marginal effect size of the first locus in an epistasis model (see additional file 1 for the description of marginal effect size) [7,8] and MAFs of both loci. For detailed derivation and equations, see reference [7]. In Model 1, the penetrance increases only when both loci have at least one minor allele. In model 2, the additional minor allele at each locus does not further increase the penetrance. Model 3 assumes that the minor allele in the first locus has marginal effect, when minor alleles in both loci are present; however, the effect is inversed. Other epistasis models are eNME models with their population prevalence ranging from 0.01 to 0.64. Their penetrances are directly cited from references [10,41,50]. Specifically, Model 4 ~ Model 7 are randomly chosen from references [10,50]; Model 8 is a ZZ model [41]; and Model 9 is an XOR model [10]. These eNME models are considered in this study since they provide a high degree of complexity to challenge ability of a method in identifying epistatic interactions.

We use different parameters to generate epistasis models. Detailed parameter settings are recorded in supplementary table S4 in additional file 1. For each model, 200 datasets are simulated each containing 2000 cases and 2000 controls. In the first 100 datasets, 100 SNPs are genotyped, while in other 100 datasets the number of genotyped SNPs is increased to 10000, which simulates high-dimensional datasets as those in GWAS. For each dataset, two SNPs are phenotype-associated, and others are phenotype-unassociated, which are set independently with MAFs chosen from [0.05, 0.5] uniformly.

In our study, parameters of each method are generally set as default. Only a few are modified according to suggestions in order to balance result accuracy and computational cost. For TEAM, permutation number is set to 100. For BOOST, interaction threshold is set to 10, i.e., results of BOOST are the epistatic interactions whose likelihood ratio test statistic values >10 with 4 degrees of freedom. For epiMODE, iteration number is set to 100.

**Figure 2:**
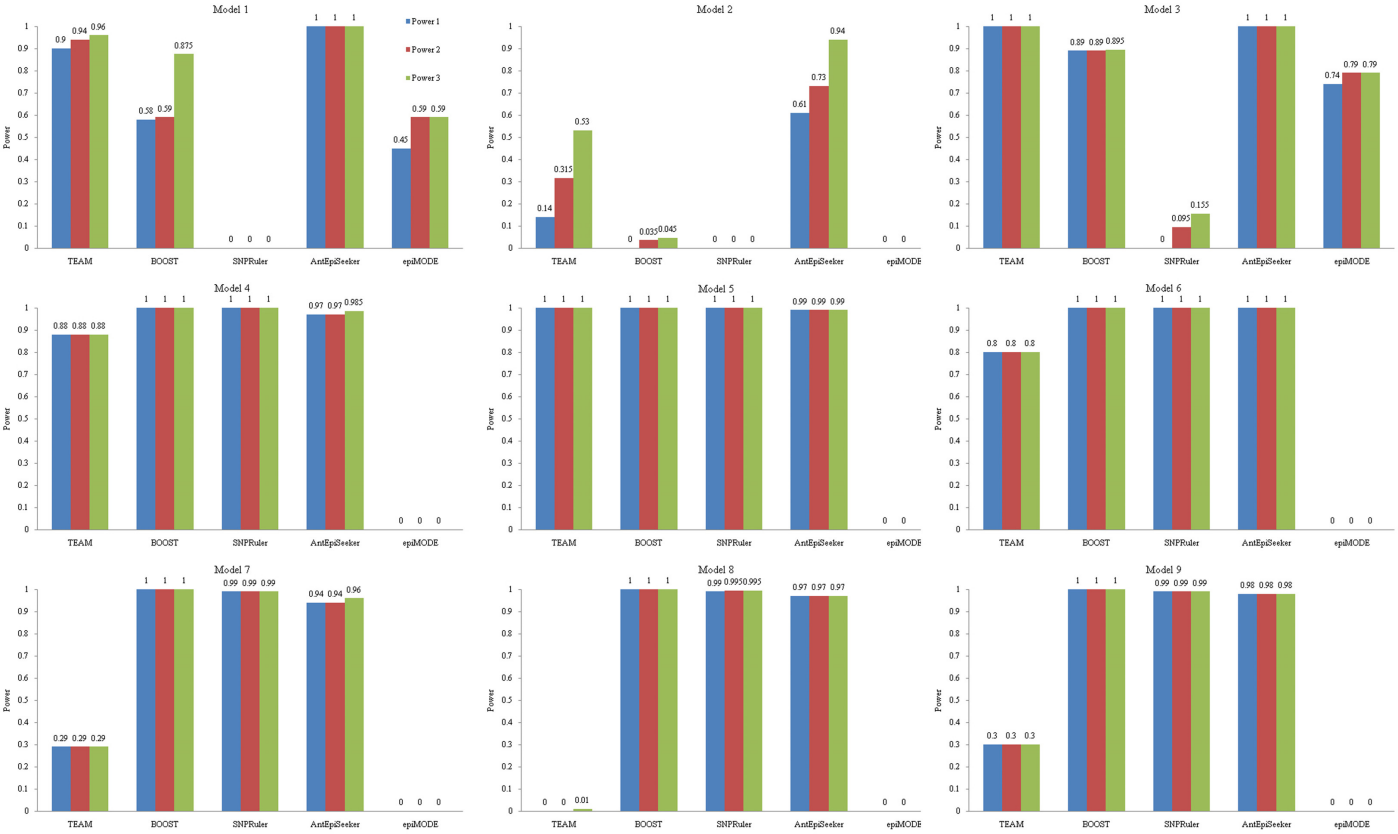
**Detection power of the methods on 100-SNP non-noise datasets**.

**Figure 3:**
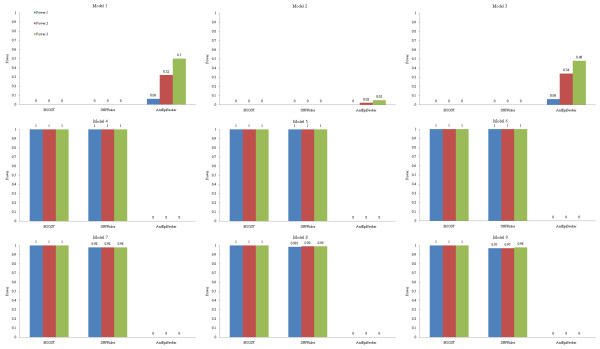
**Detection power of the methods on 10000-SNP non-noise datasets**.

Detection power of the methods on 100-SNP datasets is shown in Figure 2, and that on 10000-SNP datasets is shown in Figure 3. TEAM and epiMODE are not considered in Figure 3 due to their unaffordable computational cost on high-dimensional datasets (e.g., 10000 or more SNPs). From the figures, we have following observations.

It is seen that for both SNPRuler and BOOST, detection power on eNME models is much higher than that on eME models. Specifically, for 100-SNP datasets, they identify almost all ground-truth SNPs in eNME models. However, they have poor detection power on eME models. For 10000-SNP datasets, the situation is more serious: detection power on eME models reduces to zero. This is in consistency to the principle of them. That is, they only focus on identifying eNME models. The results are also consistent with and complementary to previous reported results [38]. In addition, both methods are based on contingency table, which reflects (direct-) dependence structure between two or more variables, and has been proven to be effective in identifying interactions [51-53].

One might believe that it is the high population prevalence of a model that makes epistasis detection of SNPRuler and BOOST easy. However, this is not the case. For example, for 100-SNP datasets, detection power on Model 3 is higher, but population prevalence is lower than those on Model 2. Hence model type may be a factor that influences detection power. In fact, from our experiment, BOOST is more sensitive to model type compared with SNPRuler.

In contrast to SNPRuler and BOOST, for 100-SNP datasets, AntEpiSeeker has good performance on both eME and eNME models. It identifies almost all ground-truth SNPs except in Model 2. For Model 2, compared with other methods, AntEpiSeeker is still a winner, though detection power does not reach a perfect level. Detection power of AntEpiSeeker decreases for 10000-SNP datasets. Specifically, it is low on eME models and zero on eNME models. This implies that the rules of ants selecting paths in AntEpiSeeker are sensitive to SNPs each has strong association with the phenotype. The factor that significantly influences the decrease of detection power from 100-SNP to 10000-SNP datasets is the inevitably increased search space: only 4950 possible two-locus interactions need to be investigated for 100-SNP datasets, while it becomes about fifty million for 10000-SNP datasets.

TEAM has good detection power on Model 3 and Model 5, but detects no ground-truth SNPs in Model 8. On other models, it has moderate detection power. These results demonstrate that TEAM is model-sensitive.

EpiMODE has the worst performance on seven models. Only on Model 1 and Model 3, has it moderate detection power.

From above analysis, it is seen that contingency table is a pathway for identifying eNME, and AntEpiSeeker provides a good search strategy for identifying both eME and eNME. Hence a new direction might be a combination of contingency table based relevance measure and AntEpiSeeker like search principle for detecting both eME and eNME. Till now, it seems that no methods can be insensitive to model types. Detecting all types of epistasis models is still a challenging task.

### Robustness Analysis

Commonly encountered noise in genetic epidemiology studies is missing data, genotyping error and phenocopy, which have been simulated and respectively added into datasets for comparison study [33,34]. In our experiments, missing data is simulated by removing 5% of genotype information randomly. Genotyping error is simulated using a directed-error model [54], so that, 5% of genotypes are selected and biased toward to one allele, unless it is already homozygous in the biased direction. Phenocopy is simulated such that 20% of cases are affected under a particular environmental condition, rather than genotype conditions. For each model, 100 datasets for each noise type are generated.

We study the robustness of methods to missing data, genotyping error and phenocopy respectively, except for that of TEAM and BOOST to missing data. This is due to the pre-process of both methods for handling missing data. For TEAM, it estimates the value of missing data using other tools, and for BOOST, it just simply removes SNPs relating to missing data.

**Figure 4:**
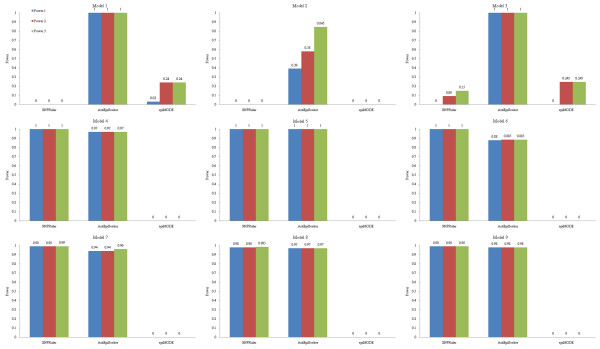
**Detection power of the methods on 100-SNP noise datasets with 5% missing data**.

**Figure 5:**
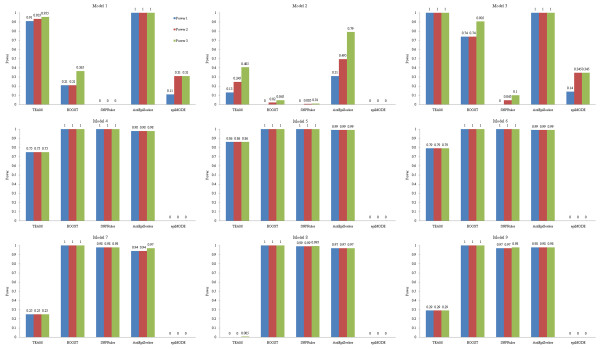
**Detection power of the methods on 100-SNP noise datasets with 5% genotyping error**.

**Figure 6:**
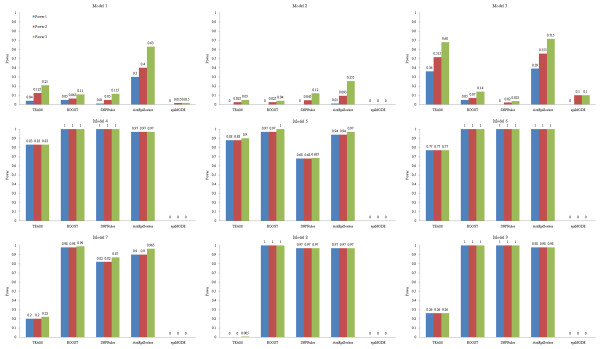
**Detection power of the methods on 100-SNP noise datasets with 20% phenocopy**.

Detection power of the methods on noise datasets with 5% missing data is shown in Figure 4, with 5% genotyping error is shown in Figure 5, and with 20% phenocopy is shown in Figure 6. DORs of the methods on noise datasets based on three forms of detection power are recorded in table 1.

**Table 1:** Degree of Robustness (DOR) values of the methods to the noise of missing data, genotyping error and phenocopy.

Noise Types	Models	Power	TEAM	BOOST	SNPRuler	AntEpiSeeker	epiMODE
Missing Data	Model 1	*power 1*	--	--	0.0000	1.0000	0.0000
		*power 2*	--	--	0.0000	1.0000	0.0000
		*power 3*	--	--	0.0000	1.0000	0.0000
	Model 2	*power 1*	--	--	0.0000	0.0000	0.0000
		*power 2*	--	--	0.0000	0.0000	0.0000
		*power 3*	--	--	0.0000	0.0433	0.0000
	Model 3	*power 1*	--	--	0.0000	1.0000	0.0000
		*power 2*	--	--	0.2925	1.0000	0.0000
		*power 3*	--	--	0.5188	1.0000	0.0000
	Model 4	*power 1*	--	--	1.0000	1.0000	0.0000
		*power 2*	--	--	1.0000	1.0000	0.0000
		*power 3*	--	--	1.0000	0.7607	0.0000
	Model 5	*power 1*	--	--	1.0000	*1.1601*	0.0000
		*power 2*	--	--	1.0000	*1.1601*	0.0000
		*power 3*	--	--	1.0000	*1.1601*	0.0000
	Model 6	*power 1*	--	--	1.0000	0.0164	0.0000
		*power 2*	--	--	1.0000	0.0214	0.0000
		*power 3*	--	--	1.0000	0.0214	0.0000
	Model 7	*power 1*	--	--	1.0000	1.0000	0.0000
		*power 2*	--	--	1.0000	1.0000	0.0000
		*power 3*	--	--	1.0000	1.0000	0.0000
	Model 8	*power 1*	--	--	0.8399	1.0000	0.0000
		*power 2*	--	--	0.8399	1.0000	0.0000
		*power 3*	--	--	0.8407	1.0000	0.0000
	Model 9	*power 1*	--	--	1.0000	1.0000	0.0000
		*power 2*	--	--	1.0000	1.0000	0.0000
		*power 3*	--	--	1.0000	1.0000	0.0000

Genotyping Error	Model 1	*power 1*	*1.1759*	0.0000	0.0000	1.0000	0.0000
		*power 2*	0.9153	0.0000	0.0000	1.0000	0.0000
		*power 3*	0.9170	0.0000	0.0000	1.0000	0.0000
	Model 2	*power 1*	0.1531	0.0000	0.0000	0.0000	0.0000
		*power 2*	0.0000	0.0000	*2.0000*	0.0000	0.0000
		*power 3*	0.0000	1.0000	*2.0000*	0.0014	0.0000
	Model 3	*power 1*	1.0000	0.0007	0.0000	1.0000	0.0000
		*power 2*	1.0000	0.0007	0.0000	1.0000	0.0000
		*power 3*	1.0000	*1.1768*	0.0000	1.0000	0.0000
	Model 4	*power 1*	0.0031	1.0000	1.0000	*1.1614*	0.0000
		*power 2*	0.0031	1.0000	1.0000	*1.1614*	0.0000
		*power 3*	0.0031	1.0000	1.0000	0.9191	0.0000
	Model 5	*power 1*	0.0051	1.0000	1.0000	1.0000	0.0000
		*power 2*	0.0051	1.0000	1.0000	1.0000	0.0000
		*power 3*	0.0051	1.0000	1.0000	1.0000	0.0000
	Model 6	*power 1*	0.8026	1.0000	1.0000	0.8415	0.0000
		*power 2*	0.8026	1.0000	1.0000	0.8415	0.0000
		*power 3*	0.8026	1.0000	1.0000	0.8415	0.0000
	Model 7	*power 1*	0.0058	1.0000	0.8399	1.0000	0.0000
		*power 2*	0.0058	1.0000	0.8399	1.0000	0.0000
		*power 3*	0.0058	1.0000	0.8399	*1.1650*	0.0000
	Model 8	*power 1*	0.0000	1.0000	1.0000	1.0000	0.0000
		*power 2*	0.0000	1.0000	1.0000	1.0000	0.0000
		*power 3*	0.0000	1.0000	1.0000	1.0000	0.0000
	Model 9	*power 1*	0.5050	1.0000	0.6862	1.0000	0.0000
		*power 2*	0.5050	1.0000	0.6862	1.0000	0.0000
		*power 3*	0.5050	1.0000	0.6862	1.0000	0.0000

Phenocopy	Model 1	*power 1*	0.0000	0.0000	*2.0000*	0.0005	0.0000
		*power 2*	0.0000	0.0000	**2.0000**	0.0027	0.0000
		*power 3*	0.0001	0.0000	**2.0000**	0.0643	0.0000
	Model 2	*power 1*	0.0000	0.0000	0.0000	0.0000	0.0000
		*power 2*	0.0000	0.1531	**2.0000**	0.0000	0.0000
		*power 3*	0.0000	0.5785	**2.0000**	0.0003	0.0000
	Model 3	*power 1*	0.0014	0.0000	0.0000	0.0023	0.0000
		*power 2*	0.0153	0.0000	0.0001	0.0261	0.0000
		*power 3*	0.1096	0.0000	0.0013	0.1542	0.0000
	Model 4	*power 1*	0.7763	1.0000	1.0000	1.0000	0.0000
		*power 2*	0.7763	1.0000	1.0000	1.0000	0.0000
		*power 3*	0.7763	1.0000	1.0000	0.9393	0.0000
	Model 5	*power 1*	0.5485	0.8808	0.1096	0.8006	0.0000
		*power 2*	0.5485	0.8808	0.1096	0.9195	0.0000
		*power 3*	0.6171	1.0000	0.1153	0.9195	0.0000
	Model 6	*power 1*	0.8513	1.0000	1.0000	1.0000	0.0000
		*power 2*	0.8513	1.0000	1.0000	1.0000	0.0000
		*power 3*	0.8513	1.0000	1.0000	1.0000	0.0000
	Model 7	*power 1*	0.1207	0.9203	0.3906	0.8315	0.0000
		*power 2*	0.1207	0.9203	0.3906	0.8315	0.0000
		*power 3*	0.2275	0.9601	0.5445	*1.0208*	0.0000
	Model 8	*power 1*	0.0000	1.0000	0.9195	1.0000	0.0000
		*power 2*	0.0000	1.0000	0.9195	1.0000	0.0000
		*power 3*	0.0124	1.0000	0.9000	1.0000	0.0000
	Model 9	*power 1*	0.5050	1.0000	*1.0403*	1.0000	0.0000
		*power 2*	0.5050	1.0000	*1.0403*	1.0000	0.0000
		*power 3*	0.5050	1.0000	*1.0403*	1.0000	0.0000

There are three types of noise added into datasets respectively. For each model with certain type of noise, each method has three DORs, since three forms of detection power are introduced. Theoretically, DORs are ranged from 0 to 1. However, realistically, there are some DORs (only a few) in table larger than 1. Most DORs with italic fonts are caused by detection power computation precision and their detection power differences are not greater than 0.01. DORs with bold fonts, which are only occurred on robustness analysis of SNPRuler to phenocopy on Model 1 and Model 2, are described and evaluated in detail.

As expected, most DORs are ranged from 0 to 1, while surprisingly, some DORs (only a few) are even larger than 1. Such a surprising finding stimulates explanation. One reason is the limited number of datasets (e.g., only 100) for detection power computation, which restricts the precision of detection power, and hence affects the DOR precision. This is the reason of most, but not all, DORs larger than 1.

Theoretically, DOR of larger than 1 indicates that detection power of a method on noise datasets is higher than that on non-noise datasets: noise might help the detection of epistasis model. As mentioned before, among the methods, some focus more on eME detection, while some focus more on eNME detection. In reality, noise might traverse an eME model to be more close to an eNME model, or vice versa. For example, the original model is an eNME model, but addition of noise biases the model to have marginal effects which makes the model more close to an eME model. This tends to possibility that the eNME model originally successfully detected by the method facilitating to eNME detection fails to be detected, leading to the DOR of the method less than 1, but be successfully detected by the method facilitating to eME detection though fails to be detected originally, leading to the DOR of the method larger than 1. We believe that this is another reason of the DOR larger than 1 for some noise data.

#### 1. Missing data

AntEpiSeeker has far better robustness than other methods on eME models, and good detection power on eNME models. This clue provides important basis for developing effective methods which possess stronger robustness to missing data. The small DORs of AntEpiSeeker on Model 2 and Model 6 implies that missing data really influences detection power, though only 5% of missing data is added into datasets. The DORs of SNPRuler that close to or even equal to 1 on most eNME models indicate that the method is robust to missing data on eNME models. EpiMODE loses its ability on all models.

#### 2. Genotyping error

TEAM has high DORs on Model 1, Model 3 and Model 6, but weak robustness on other models, which proves that its robustness is model-sensitive. As might be expected, SNPRuler has good detection power and strong robustness on eNME models, but poor ability on eME models. The DORs of BOOST are low on eME models and keep at 1 on eNME models, which is consistent to the principle of the method, i.e., it is designed specifically for eNME detection. AntEpiSeeker has high DORs on all models. EpiMODE has no ability on datasets with 5% genotyping error.

#### 3. Phenocopy

Detection power of SNPRuler is higher on two eME models (Model 1 and Model 2) with 20% phenocopy than that with no noise. This is because phenocopy traverses the eME models to be more close to eNME models, while SNPRuler only tests eNME models since eME models are pruned in the branch stage [38]. But it seems that such a traversal is not strong enough for BOOST focusing on eNME models to successfully detect them. These are the examples that noise really helps the detection of a model. Overviewing the DORs among noise types, it is seen that such extreme examples happen only for phenocopy. This is because only the phenocopy implies a real bias of the model, while other noise, i.e., missing data and genotyping error, modifies little about the model.

TEAM has high DORs on Model 4 ~Model 6, and has poor robustness on other models. This is because TEAM is sensitive to model type on its robustness. No matter what types of noise are added into datasets, BOOST always has perfect detection power and high DORs on eNME models, which implies that regression based methods are promising in detecting eNME models. Poor ability of BOOST on eME models inspires researchers to develop more effective methods based on regression strategy compared to BOOST. AntEpiSeeker has good detection power on all models, especially on eME models. Although its robustness on noise datasets needs to be improved, on viewpoint of detection power and model type dependence, AntEpiSeeker is the winner among the methods.

From above analysis, AntEpiSeeker is robust to all types of noise on eME models. Though BOOST can not handle datasets with missing data, it has perfect DORs on eNME models with either genotyping error or phenocopy. SNPRuler is robust to phenocopy on eME models and missing data on eNME models. The robustness of methods is sensitive to models and noise types. Among the methods, epiMODE is of no robustness to all the noise types.

### Sensitivity Analysis

**Figure 7:**
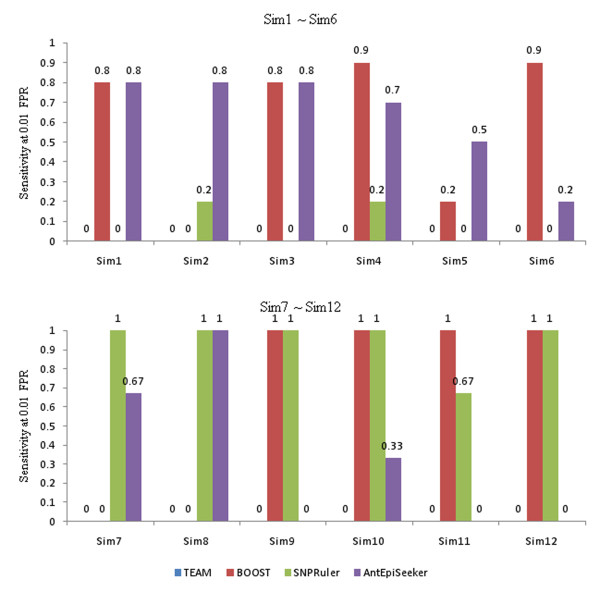
**Sensitivity of the methods at 0.01 FPR**.

**Figure 8:**
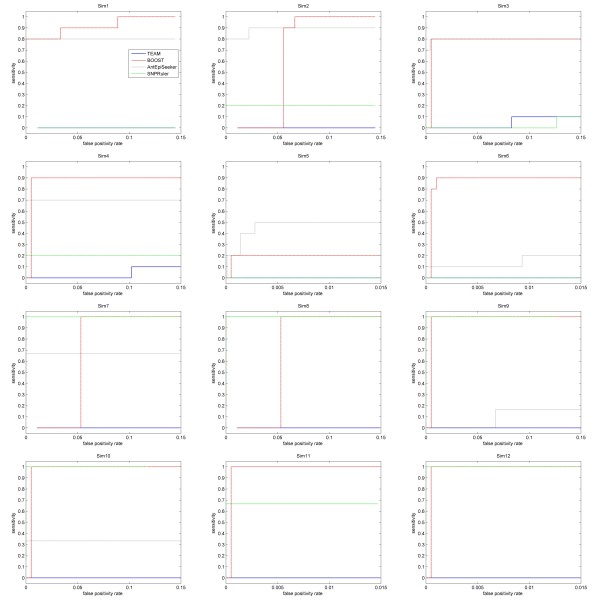
**Left-side ROC curves of the methods on datasets**.

In above experiment, a simulation dataset includes only one two-locus epistasis model. By considering that a complex disease is possibly caused by multiple epistatic interactions [4], we simulate 12 non-noise datasets (Sim1 ~ Sim12) in each of which multiple epistasis models are embedded that jointly influence the phenotype. For each of the first 6 datasets (Sim1 ~ Sim6), 6 models are embedded with 4 models being two-locus eME models and 2 models being single-locus models. Penetrances and MAFs of loci for these models are directly cited from reference [55] and shown in supplementary table S5 and supplementary table S6 in additional file 1. Other 6 datasets (Sim7 ~ Sim12) are simulated each containing 3 eNME models (Model 4 ~ Model 6). The datasets are with 2000 and 4000 samples genotyped by 100, 1000, and 10000 SNPs, respectively. Among them, Sim5, Sim6, Sim11, and Sim12 simulate high-dimensional datasets as those in GWAS. Details of these datasets are recorded in supplementary table S7 in additional file 1. Sensitivity of the methods at 0.01 FPR is given in Figure 7. Left-side ROC curves of the methods are shown in Figure 8, from which sensitivity of the methods at other FPRs can be obtained directly.

Among the methods, BOOST has the highest sensitivity at 0.01 FPR in most of the datasets, especially in Sim9 ~ Sim12. Although sensitivity of BOOST at low FPRs is zero in Sim2, Sim7 and Sim8, when reaching to 0.1 FPR, it is the winner. As expected, SNPRuler has high sensitivity in Sim7 ~ Sim12 and low in Sim1 ~ Sim6. Furthermore, by comparing ROC curves between Sim1 and Sim2, between Sim3 and Sim4, and between Sim11 and Sim12, it is seen that increasing sample size helps SNPRuler to improve detection power. For AntEpiSeeker, sensitivity at low FPRs seems drop as the number of SNPs increases, which prohibits its further application. TEAM has zero sensitivity at 0.01 FPR in all datasets. Only when reaching to 0.1 FPR, has TEAM low sensitivity in Sim3 and Sim4. EpiMODE detects nothing in all datasets and hence is not considered in this study.

From above analysis, it seems that BOOST is suitable for multiple epistasis detection. AntEpiSeeker performs well on multiple eME detection and SNPRuler performs well on multiple eME detection. Additionally, both SNPRuler and BOOST are sensitive to sample size and SNP number. TEAM and epiMODE have no ability on multiple epistasis detection.

### Computational Complexity Analysis

Experiments of the methods on Sim1 ~ Sim6 are conducted with Intel Xeon 2.00 GHz CPUs and 6 GB of RAM running Microsoft Windows XP Professional x64 Edition 2003 Service Pack 2 for computational complexity analysis. The running time of the methods on each dataset is shown in table 2.

**Table 2:** Running time (minutes) of the methods on Sim1 ~ Sim6.

Methods	Sim1	Sim2	Sim3	Sim4	Sim5	Sim6
TEAM	0.099	0.219	3.955	7.885	350.1	695.7
BOOST	0.003	0.006	0.053	0.086	3.098	4.142
SNPRuler	0.019	0.026	0.348	0.667	30.88	58.26
AntEpiSeeker	9.857	19.11	12.96	27.11	51.36	104.2
epiMODE	0.604	0.841	1607	3175	>20d*	>20d*

* represents running time is presented in days.

BOOST is the fastest among the methods. For Sim1 and Sim2, it only spends less than a second; even for datasets each with 10000 SNPs, it costs a few minutes with running time several times faster than that of other methods. This is due to its fast Boolean operation for computing contingency tables and upper bound-pruning technique [38]. Running time of SNPRuler is short, less than half an hour for sim6, and importantly, it goes up moderately. AntEpiSeeker is time affordable on handling large scale datasets. TEAM is the slowest among the methods due to its permutation test operation, although traversing minimum spanning tree helps reduce time cost. EpiMODE could not deal with datasets with 10000 SNPs at affordable time cost.

For storage requirement, TEAM, BOOST, AntEpiSeeker and epiMODE do not need much memory and run smoothly at our platform. However, SNPRuler requires unaffordable memory for large scale datasets.

From above analysis, TEAM, epiMODE and SNPRuler require either huge time cost or unaffordable memory. BOOST and AntEpiSeeker are affordable in both computation and storage requirement for large scale datasets. Hence the latter two facilitate genome-wide study in the sense.

## Conclusions

Epistasis detection helps elucidate lots of biological and biochemical pathways that underlie complex diseases of human, animal and plant. Although its computational and methodological perplexities have been well recognized, it remains a challenge in designing methods. With the tireless efforts of researchers for decades, some promising methods have been proposed. However, due to difficulties, such as lack of benchmark simulation datasets, limited epistasis models, evaluation criteria and computational burden, comparison study have not been paid much attention to. Comparison study can reveal merits and limitations of the methods and offer clues of epistasis detection to researchers, which will inspire them to develop more effective and efficient methods.

By reviewing the literature, we identify 36 methods in use for epistasis detection, and in this study, we classify them into three categories according to their search strategies, i.e., exhaustive search, stochastic search, and heuristic search. Among the methods, we select five representative methods for comparison study. They are TEAM, BOOST, SNPRuler, AntEpiSeeker and epiMODE. To do so, we need to have simulated datasets and evaluation criteria. The simulation datasets with different size, various epistasis models, absence and presence of noise are generated by a tool, epiSIM, in which the noise includes respective missing data, genotyping error and phenocopy. Three forms of detection power, robustness, sensitivity, and computational complexity are provided as evaluation criteria.

Our experimental results indicate that performance of a method varies over SNP number and sample size of datasets, epistasis models and noise types, and performance varies over methods for a dataset. Since multiple evaluation criteria are concerned, it becomes difficult to say which method is better. In terms of detection power, AntEpiSeeker performs best on eME models and BOOST performs best on eNME models. When users expect strong robustness to noise, we recommend using AntEpiSeeker, BOOST and SNPRuler. Specifically, AntEpiSeeker is robust to all types of noise on eME models; BOOST is robust to genotyping error and phenocopy on eNME models, but can not handle datasets with missing data; SNPRuler is robust to phenocopy on eME models and missing data on eNME models. In terms of sensitivity, AntEpiSeeker is the winner on eME models and both BOOST and SNPRuler perform well on eNME models. If users are conscious of computational complexity and have to handle large scale datasets, we recommend using BOOST. In terms of overall performance, AntEpiSeeker and BOOST are recommended as the efficient and effective methods. Although the use of methods usually depend on the context, according to results of this study, we sort the methods according to each criterion and give an intuitive recommendation in supplementary table S8 in additional file 1 with a number ranging from 5 (i.e., excellent) to 1 (i.e., poor).

As expected, several important conclusions can be inferred.

First, each method has its own merits and limitations, but no one is perfect. None of them are consistently better than others in all scenarios. For example, SNPRuler has perfect detection power on eNME models and spends affordable time on large scale datasets. However, it has weak detection power on eME models and requires huge memory occupancy in GWAS.

Second, a method might be superior on some models and inferior on other models, but none is insensitive to all model types. For instance, SNPRuler and BOOST have poor detection power on eME models and perfect detection power on eNME models.

Some methods, e.g., TEAM and BOOST, are limited to only two-locus epistasis detection. Some methods, e.g., SNPRuler and AntEpiSeeker, though can deal with high-order models, but with rapid growth of computational cost with interaction order.

Additionally, a method may have strong robustness on datasets with one noise type, but is weak on datasets with another noise type. For example, SNPRuler has strong robustness to missing data, but is sensitive to genotyping error.

Considering further work relating to epistasis detection, there are multiple folds.

First, epistasis models used in this study, though spreading in some sense, are still limited. More epistasis models with wider spread parameter settings should be studied. For instance, epistasis models with low population prevalence (i.e., less than 0.01) are not, but should be considered. In polygenic conditions, population prevalence of a complex disease is very low, which is one of the reasons for the meagre results from numerous GWAS.

Second, we infer that the performance may be sensitive to MAFs, linkage disequilibrium and penetrances and their impact to epistasis detection should be studied.

Furthermore, other noise such as genetic heterogeneity should be considered.

At present, epistasis models are more or less speculative and have weak biological theories, hence models based on biological systems need to be well defined for assessing a method. Furthermore, detection methods are generally based on statistical calculation, which is far too simplistic, as diseases are not a single entity, but heterogeneous conditions basically determined by the composite genotype in a network of genetic interactions, subsequently possibly modified by non-genetic factors. How to explore the wide spectrum of all biologically authentic epistasis including both eME and eNME from a genome wide scale dataset at a computationally affordable cost is a challenging task for bioinformatics researchers. From this view point, an assessment of biological relevance of epistasis models and detection methods would be highly appreciated.

EpiSIM simulator and 100-SNP non-noise datasets are available and can be downloaded from the link, https://sourceforge.net/projects/episimsimulator/files/. Other datasets, like 100-SNP noise datasets, 10000-SNP non-noise datasets and Sim1 ~ Sim12, can be obtained by contacting the corresponding authors.

## Authors' contributions

JS and JZ jointly contributed to the design of the study. JS designed and implemented the epiSIM method as well as performed the experiments, JS and JZ jointly drafted the manuscript. YS gave some statistical and computational advices to the work, and participated in designing evaluation criteria. DL, DY, and YY contributed to data analysis. All authors read and approved the final manuscript.

## Competing interests

The authors declare that they have no competing interests.

## Supplementary Material

Additional file 1 **Supplementary file for the main text**. The file is a PDF document, including a technical term description and 8 tables. Marginal effect size appeared in main text is described in detail. Table S1 and table S2 are overviews of the methods for epistasis detection. Table S3 ~ S6 record 15 disease models (2 single-locus models and 13 two-locus models). Table S7 shows information of datasets, each of which is added into multiple disease models. Table S8 is an intuitive impression of the methods. Click here for file
