# Supplementary material for: Performance analysis of novel methods for detecting epistasis
Source: BMC Bioinformatics. 2011 Dec 15;12:475. doi: 10.1186/1471-2105-12-475 (PMC3259123; doi:10.1186/1471-2105-12-475)
Supplement: Additional file 1 — Supplementary file for the main text. The file is a PDF document, including a technical term description and 8 tables. Marginal effect size appeared in main text is described in detail. Table S1 and table S2 are overviews of the methods for epistasis detection. Table S3 ~ S6 record 15 disease models (2 single-locus models and 13 two-locus models). Table S7 shows information of datasets, each of which is added into multiple disease models. Table S8 is an intuitive impression of the methods. [file 1471-2105-12-475-S1.PDF]

Performance analysis of novel methods for detecting epistasis - Supplementary file

Junliang Shang[1*], Junying Zhang[1*], Yan Sun[2], Dan Liu[1], Daojun Ye[1], Yaling Yin[1,3]

[1]School of Computer Science & Technology, Xidian University, Xi'an 710071, China

[2]Shannxi people's fine arts publishing house, Xi'an 710003, China

[3]Information School, Xi'an Economical and Financial University, Xi'an 710100, China

*Corresponding author

Email addresses:

JS - jlshang@mail.xidian.edu.cn

JZ - jyzhang@mail.xidian.edu.cn

YS - sunyan225@126.com

DL - liudan8709@yahoo.com.cn

DY - bruce1013@163.com

YY - dir_yyll@126.com

Description of marginal effect size

Here we describe a technical term, known as marginal effect size (MES), which is used in main text to calculate penetrances of two-locus epistasis models displaying marginal effects.

MES is defined as the marginal effect of the heterozygous genotype at the first locus in an epistasis model, i.e., the marginal effect of genotype Aa to AA, which is written as $\lambda_1 = \frac{p\left(D|Aa\right)}{p\left(\bar{D}|Aa\right)} \Big/ \frac{p\left(D|AA\right)}{p\left(\bar{D}|AA\right)} - 1$, where $D/\bar{D}$ denote the affected/unaffected status. Marginal odds of AA and Aa are given as

$$\frac{p\left(D|AA\right)}{p\left(\bar{D}|AA\right)} = \frac{p\left(D|AABB\right)\cdot p\left(BB\right) + p\left(D|AABb\right)\cdot p\left(Bb\right) + p\left(D|AAbb\right)\cdot p\left(bb\right)}{p\left(\bar{D}|AABB\right)\cdot p\left(BB\right) + p\left(\bar{D}|AABb\right)\cdot p\left(Bb\right) + p\left(\bar{D}|AAbb\right)\cdot p\left(bb\right)},$$

$$\frac{p\left(D|Aa\right)}{p\left(\bar{D}|Aa\right)} = \frac{p\left(D|AaBB\right)\cdot p\left(BB\right) + p\left(D|AaBb\right)\cdot p\left(Bb\right) + p\left(D|Aabb\right)\cdot p\left(bb\right)}{p\left(\bar{D}|AaBB\right)\cdot p\left(BB\right) + p\left(\bar{D}|AaBb\right)\cdot p\left(Bb\right) + p\left(\bar{D}|Aabb\right)\cdot p\left(bb\right)}.$$

For simulating two-locus epistasis models, two parameters, baseline penetrance α and relative penetrance f, are used and need to be determined (see supplementary table S3). $p\left(D|\cdot\right)$ in above formula are hence expressed by α and f. Given MES λ_1, MAFs of the loci (A and B) and population prevalence $p\left(D\right)$, the solution of α and f can easily be obtained [1], where $p\left(D\right)$ is written as

$$\begin{aligned} p\left(D\right) = {} & p\left(D|AABB\right)\cdot p\left(AABB\right) + p\left(D|AABb\right)\cdot p\left(AABb\right) + p\left(D|AAbb\right)\cdot p\left(AAbb\right) \\ & + p\left(D|AaBB\right)\cdot p\left(AaBB\right) + p\left(D|AaBb\right)\cdot p\left(AaBb\right) + p\left(D|Aabb\right)\cdot p\left(Aabb\right) \\ & + p\left(D|aaBB\right)\cdot p\left(aaBB\right) + p\left(D|aaBb\right)\cdot p\left(aaBb\right) + p\left(D|aabb\right)\cdot p\left(aabb\right) \end{aligned}.$$

Although it is a popular way to simulate two-locus epistasis models and has been widely used in reference [1-9], it still has three limitations.

First, this method simulates restricted models in which the penetrances are specified by just two parameters, i.e., α and f. However, in fact there are nine penetrances in a general two-locus epistasis models.

Second, it considers the marginal effect of genotype Aa to AA (λ_1) and misses other marginal effects, including the marginal effect of genotype aa to AA (λ_2), the marginal effect of genotype Bb to BB (λ_3), the marginal effect of genotype bb to BB (λ_4). Hence it can not guarantee to simulate epistasis models that display no marginal effects.

Third, it is only a method to simulate two-locus epistasis models and difficult to be generalized for simulating high-order epistasis models.

The use of this method in our study is based on two reasons. First, till now, there are no better methods in simulating such epistasis models. Second, previous reported results of many comparison studies [3-7] are generally based on this model-simulation method. For comparison fair, we use it.

In our study, only penetrances of the first three models, i.e., model 1 ~ model 3 shown in supplementary table S3, are determined using this method. They are epistasis models displaying marginal effects. For other models displaying no marginal effects, i.e., model 4 ~ model 9 in supplementary table S4, their penetrances are directly cited from reference [10-12], since finding such type of models is a challenging “dark area”. In other words, penetrances of these models are not determined by this method.

Since four marginal effects of a two-locus epistasis model, discussed above, may have a strong influence on detection power of different methods, they are recorded in supplementary table S4.

Table S1. Details of the methods for detecting epistasis.

Strategies	Methods	Articles	Journals	Year	Links
Exhaustive	Combinatorial Partitioning Method (CPM)	A combinatorial partitioning method to identify multilocus genotypic partitions that predict quantitative trait variation	Genome research	2001	--
	Multifactor Dimensionality Reduction (MDR)	Multifactor-Dimensionality Reduction Reveals High-Order Interactions among Estrogens-Metabolism genes in sporadic breast cancer	American journal of human genetics	2001	http://sourceforge.net/projects/mdr/
	Restricted Partitioning Method (RPM)	Detecting epistatic interactions contributing to quantitative traits	genetic epidemiology	2004	--
	Full Interaction Model (FIM)	Genome-wide strategies for detecting multiple loci that influence complex diseases	Nature genetics	2005	http://www.cbil.ece.vt.edu/ResearchOngoingSNP.htm
	Information Gain (IG)	A flexible computational framework for detecting, characterizing, and interpreting statistical patterns of epistasis in genetic studies of human disease susceptibility	Journal of theoretical biology	2006	http://www.cbil.ece.vt.edu/ResearchOngoingSNP.htm
	Focused Interaction Testing Framework (FITF)	A testing framework for identifying susceptibility genes in the presence of epistasis	American journal of human genetics	2006	http://hydra.usc.edu/fitf
	1-degree-of-freedom	Powerful multilocus tests of genetic association in the presence of gene-gene and gene-environment interactions	American journal of human genetics	2006	--
	Backward Genotype-Trait Association (BGTA)	Backward genotype-trait association (BGTA)- based dissection of complex traits in case-control design	human heredity	2006	http://statgene.stat.columbia.edu
	FastANOVA	FastANOVA: an efficient algorithm for genome-wide association study	KDD'08	2008	http://www.csbio.unc.edu/epistasis/
	COE	COE:A general approach for efficient genome-wide two-locus epistasis test in disease association study	Molecular biology	2009	http://www.csbio.unc.edu/epistasis/download.php
	FastChi	FastChi: an effcient algorithm for analyzing gene-gene interactions	Pacific Symposium on Biocomputing	2009	http://www.csbio.unc.edu/epistasis/download.php

	BOolean Operation-based Screening and Testing (BOOST)	BOOST: A Fast Approach to Detecting Gene-Gene Interactions in Genome-wide Case-Control Studies	American journal of human genetics	2010	http://bioinformatics.ust.hk/BOOST.zip
	Bayesian Network Minimum Bit Length score (BNMBL)	Identifying genetic interactions from genome-wide data using Bayesian networks	Genetic Epidemiology	2010	--
	Tree-based Epistasis Association Mapping (TEAM)	TEAM: efficient two-locus epistasis tests in human genome-wide association study	Bioinformatics	2010	http://www.csbio.unc.edu/epistasis/download.php
Stochastic	Classification and Regression Trees (CART)	Classification and Regression Trees	*	1984	http://cran.r-project.org/web/packages/tree/index.html
	Random Forests	Machine Learning	*	2001	http://cran.r-project.org/web/packages/randomForest/index.html
	Multivariate Adaptive Regression Spline (MARS)	Tree and spline based association analysis of gene-gene interaction models for ischemic stroke	statistics in medicine	2004	http://lib.stat.cmu.edu/
	Monte Carlo Logic Regression	Identifying Interacting SNPs using Monte Carlo Logic Regression	Genetic Epidemiology	2005	http://cran.r-project.org/web/packages/LogicReg/index.html
	Genetic Programming optimized Neural Network (GPNN)	GPNN: power studies and applications of a neural network method for detecting gene-gene interactions in studies of human disease	BMC bioinformatics	2006	--
	Bayesian Epistasis Association Mapping (BEAM)	Bayesian inference of epistatic interactions in case-control studies	Nature genetics	2007	http://www.people.fas.harvard.edu/~junliu/BEAM/
	HapForest	A forest-based approach to identifying gene and gene-gene interactions	PNAS	2007	http://c2s2.yale.edu/software packages/HapForest/

	LogicFS	Identification of SNP interactions using logic regression	Biostatistics	2008	http://bioconductor.org/packages/2.4/bioc/html/logicFS.html
	detection of EPIstatic interactions using random FOREST (epiForest)	A random forest approach to the detection of epistatic interactions in case-control studies	BMC Bioinformatics	2009	http://bioinfo.au.tsinghua.edu.cn/epiForest
	MegaSNPHunter	MegaSNPHunter: a learning approach to detect disease predisposition SNPs and high level interactions in genome wide association study	BMC Bioinformatics	2009	http://bioinformatics.ust.hk/MegaSNPHunter.html
	epistatic MOdule DEtection (epiMODE)	Epistatic Module Detection for Case-Control Studies: A Bayesian Model with a Gibbs Sampling Strategy	PLOS genetics	2009	http://bioinfo.au.tsinghua.edu.cn/epiMODE/
	Random Jungle	On safari to Random Jungle: a fast implementation of Random Forests for high-dimensional data	Bioinformatics	2010	http://www.randomjungle.org/rjungle/rjunglenews
Heuristic	Trimming, Weighting and Grouping (TWG)	Trimming, weighting, and grouping SNPs in human case-control association studies	Genome research	2001	http://linkage.rockefeller.edu/ott/sumstat.html
	Two-stage Two-locus	Two-stage Two-locus models in genome-wide association	PLOS genetics	2006	--
	Penalized Logistic Regression (PLR)	Penalized logistic regression for detecting gene interactions	Biostatistics	2007	--
	INTERSNP	INTERSNP: genome-wide interaction analysis guided by a priori information	Bioinformatics	2009	http://intersnp.meb.uni-bonn.de/
	Association Graph Reduction (AGR)	Methods for detecting multi-locus genotype-phenotype association	Rice University	2009	--
	RandomPat	Detecting disease-associated genotype patterns	BMC Bioinformatics	2009	http://www.genemapping.cn
	SNPHarvester	SNPHarvester: a filtering-based approach for detecting epistatic interactions in genome-wide association studies	Bioinformatics	2009	http://bioinformatics.ust.hk/SNPHarvester.html

Maximum Entropy Conditional Probability Modelling (MECPM)	An algorithm for learning maximum entropy probability models of disease risk that efficiently searches and sparingly encodes multilocus genomic interactions	Bioinformatics	2009	http://www.cbil.ece.vt.edu/ResearchOngoingSNP.htm
AntEpiSeeker	AntEpiSeeker: detecting epistatic interactions for case-control studies using a two-stage ant colony optimization algorithm	BMC research notes	2010	http://nce.ads.uga.edu/~romdhane/AntEpiSeeker/index.html
SNPRuler	Predictive rule inference for epistatic interaction detection in genome-wide association studies	Bioinformatics	2010	http://bioinformatics.ust.hk/SNPRuler.zip

"*": it is a general method that generalizes to detect epistasis; "--": it does not provide software package.

Table S2. Main similarities/differences among the methods.

Features	TEAM	BOOST	SNPRuler	AntEpiSeeker	epiMODE
Exhaustive Search	√	√	×	×	×
Stochastic Search	×	×	×	×	√
Heuristic Search	×	×	√	√	×
Multi-Stage	×	√	√	√	×
Contingence Table	√	√	√	×	×
Regression Model	×	√	×	×	×
Permutation Test	√	×	×	×	√
Chi-square Test	×	√	√	√	√
Tree Structure	√	×	√	×	×
Bonferroni Correction	×	√	√	√	√
Interactive Effect	√	√	√	√	√
Main/Marginal Effect	√	×	×	√	√
Full Effect	√	×	×	√	√

For an epistasis model, full effect consists of marginal effects and interactive effects. AntEpiSeeker, TEAM and epiMODE focus on full effect of a model. BOOST and SNPRuler only test interactive effect of an epistasis model.

Table S3. Penetrance functions of the first three models (Model 1 ~ Model 3).

Models	Genotypes of locus A	Genotypes of locus B		
		BB	Bb	bb
Model 1	AA	α	α	α
	Aa	α	$\alpha(1+f)^2$	$\alpha(1+f)^3$
	aa	α	$\alpha(1+f)^3$	$\alpha(1+f)^4$
Model 2	AA	α	α	α
	Aa	α	$\alpha(1+f)$	$\alpha(1+f)$
	aa	α	$\alpha(1+f)$	$\alpha(1+f)$
Model 3	AA	α	α	α
	Aa	αf	α/f	α/f
	aa	αf	α/f	α/f

α is the baseline penetrance and f is the relative penetrance. Both parameters can be determined given population prevalence, marginal effect size of the first locus in model and MAFs of both loci (see description of marginal effect size).

Table S4. Details of the models Model 1 ~ Model 9.

Models	MAF (a)	MAF (b)	Prevalence	λ_1	λ_2	λ_3	λ_4	Genotypes (locus A)	Penetrance tables		
									Genotypes (locus B)		
									BB	*Bb*	*bb*
Model 1	0.3	0.2	0.100	0.297	0.518	0.445	0.775	*AA*	0.087	0.087	0.087
								Aa	0.087	0.146	0.190
								aa	0.087	0.190	0.247
Model 2	0.4	0.4	0.050	0.306	0.306	0.306	0.306	*AA*	0.042	0.042	0.042
								Aa	0.042	0.061	0.061
								aa	0.042	0.061	0.061
Model 3	0.4	0.2	0.010	0.166	0.166	-0.390	-0.390	*AA*	0.009	0.009	0.009
								Aa	0.013	0.006	0.006
								aa	0.013	0.006	0.006
Model 4	0.2	0.2	0.640	0.000	0.000	0.001	0.002	*AA*	0.486	0.960	0.538
								Aa	0.947	0.004	0.811
								aa	0.640	0.606	0.909
Model 5	0.5	0.5	0.300	0.000	0.012	0.024	0.012	*AA*	0.470	0.230	0.270
								Aa	0.240	0.270	0.420
								aa	0.240	0.440	0.090
Model 6	0.4	0.4	0.170	0.000	0.001	-0.001	0.000	*AA*	0.068	0.299	0.017
								Aa	0.289	0.044	0.285
								aa	0.048	0.262	0.174

Model 7	0.4	0.4	0.087	0.005	0.005	0.000	-0.004	*AA*<br>*Aa*<br>*aa*	0.103<br>0.098<br>0.021	0.063<br>0.086<br>0.147	0.124<br>0.069<br>0.059
Model 8	0.5	0.5	0.038	0.000	0.000	0.000	0.000	*AA*<br>*Aa*<br>*aa*	0.000<br>0.000<br>0.100	0.000<br>0.050<br>0.000	0.100<br>0.000<br>0.000
Model 9	0.5	0.5	0.010	0.000	0.000	0.000	0.000	*AA*<br>*Aa*<br>*aa*	0.000<br>0.020<br>0.000	0.020<br>0.000<br>0.020	0.000<br>0.020<br>0.000

λ_1 is the marginal effect of genotype Aa to AA; λ_2 is the marginal effect of genotype aa to AA; λ_3 is the marginal effect of genotype Bb to BB; λ_4 is the marginal effect of genotype bb to BB.

Table S5. Single-locus models in Sim1 ~ Sim6.

**Models**	**Parameters**	*AA*	*Aa*	*aa*
Model 10	Penetrance	0.00	0.50	0.50
	MAF (*a*)		0.10	
Model 11	Penetrance	0.00	0.00	0.50
	MAF (*a*)		0.40	

Table S6. Two-locus models in Sim1 ~ Sim6.

Models	MAF (*a*)	MAF (*b*)	Genotypes (locus *A*)	Penetrance tables		
				Genotypes (locus *B*)		
				BB	*Bb*	*bb*
Model 12	0.20	0.30	*AA*	0.0	0.0	0.0
			Aa	0.0	0.75	0.75
			aa	0.0	0.75	0.75
Model 13	0.25	0.25	*AA*	0.1	0.1	0.0
			Aa	0.1	0.1	0.0
			aa	0.0	0.0	0.0
Model 14	0.20	0.20	*AA*	0.0	1.0	1.0
			Aa	1.0	1.0	1.0
			aa	1.0	1.0	1.0
Model 15	0.40	0.25	*AA*	0.0	0.0	0.0
			Aa	0.0	0.5	1.0
			aa	0.0	1.0	1.0

Table S7. Details of the datasets Sim1 ~ Sim12.

Datasets	Cases	Controls	SNPs
Sim1	1000	1000	100
Sim2	2000	2000	100
Sim3	1000	1000	1000
Sim4	2000	2000	1000
Sim5	1000	1000	10000
Sim6	2000	2000	10000
Sim7	1000	1000	100
Sim8	2000	2000	100
Sim9	1000	1000	1000
Sim10	2000	2000	1000
Sim11	1000	1000	10000
Sim12	2000	2000	10000

Table S8 - An intuitive impression of compared methods.

Criteria/noise types	Models	TEAM	BOOST	SNPRuler	AntEpiSeeker	epiMODE
Power/non-noise	eME	4	3	1	5	2
	eNME	2	5	4	3	1
Power/missing data	eME	2	2	3	5	4
	eNME	2	2	5	4	3
Power/genotyping error	eME	4	3	1	5	2
	eNME	2	5	4	3	1
Power/phenocopy	eME	4	3	2	5	1
	eNME	2	5	3	4	1
Robustness/missing data	eME	2	2	4	5	3
	eNME	2	2	5	4	3
Robustness/genotyping error	eME	4	2	3	5	1
	eNME	2	5	3	4	1
Robustness/phenocopy	eME	3	2	5	4	1
	eNME	2	5	3	4	1
Sensitivity/non-noise	eME	2	4	3	5	1
	eNME	2	4	5	3	1
Computational cost	both	2	5	4	3	1

The number range from 5 (i.e., excellent) to 1 (i.e., poor).

Reference

1. Zhang Y, Liu JS: **Bayesian inference of epistatic interactions in case-control studies**. *Nat Genet* 2007, **39**(9):1167-1173.
2. Marchini J, Donnelly P, Cardon LR: **Genome-wide strategies for detecting multiple loci that influence complex diseases**. *Nat Genet* 2005, **37**(4):413-417.
3. Tang W, Wu X, Jiang R, Li Y: **Epistatic module detection for case-control studies: a Bayesian model with a Gibbs sampling strategy**. *PLoS Genet* 2009, **5**(5):e1000464.
4. Wang Y, Liu G, Feng M, Wong L: **An empirical comparison of several recent epistatic interaction detection methods**. *Bioinformatics* 2011, **27**(21):2936-2943.
5. Wan X, Yang C, Yang Q, Xue H, Tang NL, Yu W: **Predictive rule inference for epistatic interaction detection in genome-wide association studies**. *Bioinformatics* 2010, **26**(1):30-37.
6. Wan X, Yang C, Yang Q, Xue H, Fan X, Tang NL, Yu W: **BOOST: A fast approach to detecting gene-gene interactions in genome-wide case-control studies**. *Am J Hum Genet* 2010, **87**(3):325-340.
7. Wang Y, Liu X, Robbins K, Rekaya R: **AntEpiSeeker: detecting epistatic interactions for case-control studies using a two-stage ant colony optimization algorithm**. *BMC Res Notes* 2010, **3**:117.
8. Yang C, He Z, Wan X, Yang Q, Xue H, Yu W: **SNPHarvester: a filtering-based approach for detecting epistatic interactions in genome-wide association studies**. *Bioinformatics* 2009, **25**(4):504-511.
9. Wan X, Yang C, Yang Q, Xue H, Tang NL, Yu W: **MegaSNPHunter: a learning approach to detect disease predisposition SNPs and high level interactions in genome wide association study**. *BMC Bioinformatics* 2009, **10**:13.
10. Li W, Reich J: **A complete enumeration and classification of two-locus disease models**. *Hum Hered* 2000, **50**(6):334-349.
11. Frankel WN, Schork NJ: **Who's afraid of epistasis?** *Nat Genet* 1996, **14**(4):371-373.
12. Velez DR, White BC, Motsinger AA, Bush WS, Ritchie MD, Williams SM, Moore JH: **A balanced accuracy function for epistasis modeling in imbalanced datasets using multifactor dimensionality reduction**. *Genet Epidemiol* 2007, **31**(4):306-315.
